# Adverse event reports in patients taking psychiatric medication during pregnancy from spontaneous reports in Japan and the United States: an approach using latent class analysis

**DOI:** 10.1186/s12888-020-02525-z

**Published:** 2020-03-12

**Authors:** Tatsuhiko Anzai, Kunihiko Takahashi, Michiko Watanabe, Mayumi Mochizuki, Atsuko Murashima

**Affiliations:** 1 Keio University Graduate School of Health Management, 4411 Endo, Fujisawa-shi, Kanagawa 252-0883 Japan; 2 Statistics Analysis Department 1, EPS Corporation, 6-85 Shinogawa-machi, Shinjuku-ku, Tokyo, 162-0815 Japan; 3 Department of Biostatistics, Nagoya University Graduate School of Medicine, 65 Tsurumai-cho, Showa-ku, Nagoya, 466-8550 Japan; 4 Keio University Faculty of Pharmacy, 1-5-30 Shibakoen, Minato-ku, Tokyo, 105-8512 Japan; 5 Japan Drug Information Institute in Pregnancy (J-TIS), National Center for Child Health and Development, 1-5-30 Shibakoen, Minato-ku, Tokyo, 105-8512 Japan

**Keywords:** pregnancy, adverse events, psychiatric medication, spontaneous reports, latent class analysis, polypharmacy

## Abstract

**Background:**

Little is known regarding the association between adverse events (AEs) and psychiatric medications administered to pregnant women in clinical trials during the pre-marketing period. This study analyzes reports of AE association with psychiatric medication administrated during pregnancy using post-marketing spontaneous reports of AE from the Japanese Adverse Drug Event Report (JADER) database and Food and Drug Administration Adverse Event Reporting System in the United States (FAERS-US).

**Methods:**

We summarized AE reports of psychiatric medication administrated during pregnancy by comparing data obtained from JADER and FAERS-US databases with medication patterns determined as classes via latent class analysis. The odds ratios (ORs) of AE reports categorized into system organ classes in which each class was compared with those without psychiatric medications.

**Results:**

The proportions of AE reports under psychiatric medication in pregnancy among all AE reports were 22.0% and 16.6% in JADER and FAERS-US, respectively. The 10,389 reports of psychiatric medication during pregnancy were classified into 11 classes. The proportion of patients receiving four or more psychiatric drugs in JADER was larger than that in FAERS-US. The maximum number of reports in combinations of AE and medication pattern in JADER was 169, for ‘general disorders and administration site conditions’ from the class of four or more medications (OR = 9.1), while that in FAERS-US was 1,654, for ‘injury, poisoning, and procedural complications’ from the class of single psychiatric medication (OR = 2.8).

**Conclusions:**

The main AE reports and associated AE differed depending on medication patterns in pregnant women taking psychiatric medication. This study may provide a prediction of AEs that are likely to be reported with each medication pattern. Our findings of the association between AE reports and medication patterns could help improve the administration of psychiatric medications during pregnancy, though further research on additional datasets is needed to clarify these results.

## Background

Recent studies have shown that maternal psychiatric disorders during pregnancy and the postpartum are associated with negative effects on offspring, including maladaptive fetal growth and development as well as overall health [1–3]. The number of women who reported mental health challenges, such as depression, and the number of pregnant women who take psychiatric medication has been increasing [4, 5]. For example, approximately 13% of women in the United States (US) were treated with antidepressants during pregnancy in 2003, which is twice the figure reported in 1999 [6], and 15.4% of childbearing age women received antidepressants in 2013 [7]. On the other hand, psychotropic medication polypharmacy, i.e. use of more than one medication simultaneously, is common and, in some patient groups, may have increased in recent years [8]. In particular, combination therapy of psychiatric medication is more commonly used in Japan than the US [9]. Although the use of this combination therapy in patients during pregnancy is of concern, the actual patterns of treatment with psychiatric medication in patients during pregnancy have not yet been investigated enough. Moreover, medication patterns for psychiatric disorders could differ for each country.

Medications used during pregnancy must be administrated with caution as the therapeutic desire must be weighed against the potential risks of adverse perinatal and postnatal outcomes, for both the women and the fetus [10]. Information regarding the safety of psychiatric medication is typically included in clinical trials during the pre-marketing period [11]. However, since pregnant women are often excluded from clinical trials, information regarding adverse events associated with these medications during pregnancy is limited [10]. Therefore, pharmacoepidemiologic studies are essential to evaluate the safety and efficacy of these drugs in an environment different from that of controlled clinical trials [12]. In particular, pharmacovigilance refers to the science and activities related to the collection, detection, assessment, monitoring, and prevention of adverse events (AEs) in pharmaceutical products, with a focus on safety surveillance and risk management post-marketing [13]. AE reports are a cornerstone of pharmacovigilance and are therefore, closely monitored by regulatory authorities [14]. For the purpose of pharmacovigilance, such reports are systematically collected through spontaneous reporting systems such as the Japanese Adverse Drug Event Report (JADER) database and the Food and Drug Administration Adverse Event Reporting System (FAERS) in the US. These reporting systems play an important role in pharmacovigilance by providing information from clinical settings throughout the lifetime of a drug [12].

Recently, several studies have expressed specific safety concerns using data from spontaneous AE reports from real clinical practice, e.g. assessing the relationship between AEs and combinations of psychiatric medications [15], as well as relationship between cardiovascular AEs and antidepressants [16]. However, despite the importance of the safety of medication use during pregnancy, AE reports in pregnant women receiving psychiatric medication have not been adequately evaluated. The information of medication pattern in pregnant women receiving psychiatric medication who reported any AEs can help to understand not only AEs but also to stratify characteristics from the reports and their differences in the countries where the reports were generated. Furthermore, although some limitations exist in assessing outcomes from spontaneous reports [12], this evaluation of AEs allows the quantification of the possible associations between AEs and psychiatric medications.

In this study, we summarized and compared the characteristics of pregnant women reporting AEs of psychiatric medication from Japan and the US, and from spontaneous reports, we detected medication patterns in pregnant women receiving psychiatric medication who reported any AEs. The latent class analysis (LCA) was applied to detect the medication patterns of AE reports. LCA is a known statistical method that enables the grouping of individuals into one or more distinct classes on the basis of responses to a finite number of indicators and has been used widely in medical research analysis [17, 18]. The classification in this analysis was based on medication administrated during pregnancy (including number of drugs and specific combinations of drugs) to identify subgroups of people who had similar patterns of medication usage. Moreover, we assessed possible associations between reports of AEs and medications, though it did not directly estimate the risk of AEs. LCA Classification was also expected to compensate for the instability of performing calculations (e.g. odds ratio) based on the few numbers of reports for each drug-AE combination. Through this approach, we sought to reveal the differences of AE reports and their associated medication patterns in pregnant women receiving psychiatric medication between the two countries. We also quantified the relationship between reports of AEs and medication patterns, and compared these results to those gleaned from AE reports of patients receiving medications other than psychiatric ones during pregnancy.

## Methods

We analyzed two sets of data from post-marketing spontaneous pharmacovigilance databases: the JADER database, which was created by the Pharmaceuticals and Medical Devices Agency, Japan, and FAERS, which was created by the US Food and Drug Administration (FDA). The JADER database contains over 440,000 AE cases occurred specifically in Japan and FAERS includes over 14,000,000 cases of AE reported after April 2004. Spontaneous reports are reports of an adverse event by physicians, pharmacists, other healthcare professionals, manufacturers, and consumers, which are sent to regulatory agencies. The source of the database is compliant with the International Conference for Harmonisation (ICH) of Technical Requirements for Registration of Pharmaceuticals for Human Use Guidelines, and the databases adhere to ICH-standardized AE information guidelines [19, 20]. The databases provide the main items for each AE case, such as age, sex, medicinal product/substance name, nature of AE, and case outcome. Multiple drug names in polypharmacy, multiple AEs, and multiple medical histories are allowed in cases. The contributions of the AE of medications provided were classified into three categories: ‘suspected medicine’, ‘concomitant medicine’, and ‘interaction’. The structures of the databases are shown elsewhere [15, 20].

The period of data collected from JADER ranged from April 2004 through December 2018, while that from FAERS ranged from July 2014 through December 2018, since information regarding the age group designated ‘neonatal’ was not available from FAERS prior to July 2014. The JADER contains AE cases occurring specifically in Japan, whereas the cases occurring in the US were selected from FAERS (FAERS-US).

We selected AE cases under any medication used during pregnancy and categorized them as either psychiatric medication or not as follows. First, to identify candidates for analysis, we selected reports that matched the inclusive criteria (see Sakai et al. [21] for details): (1) reported AE belonging to six standardized medical dictionary definitions of regulatory activities (MedDRA) queries (SMQs) related to pregnancy (the list of SMQs is specified in Additional file 1: Table S1); (2) disease or indication for medication use belonging to first three SMQs. Here, reported AE, disease, and indication of medications were coded using MedDRA 21.1. Among the reports, candidates of the ages of 10–49 years were included. Additionally, we included neonatal patient reports that identified age group in JADER and in FAERS-US as well as those who experienced trans-placental exposure. Finally, reports for medications whose disease/indication for use (not events) was recorded as a congenital anomaly or neonatal disorder were excluded from the dataset. To classify reports pertaining to patients receiving psychiatric medication, the drug name in the standard commodity classification field belonged to ‘Hypnotics and sedatives, antianxietics’, ‘Antiepileptics’, ‘Antiparkinsonians’ agents’, and/or ‘Psychotropic agents’ in JADER. Antiepileptics were included as psychiatric medication since antiepileptic drugs are commonly used to treat psychiatric disorders [22]; in fact, several issues of antiepileptic drug use in patients with psychiatric disorder were discussed in Japan [23]. For FAERS-US data, such reports were converted to the same format as JADER using a conversion table (WHO-DD drug name to Iyakuhinmei data file) coded via the ‘Cross Reference Tool Japan’. Cases with multiple drug names regardless of the contribution of each drug to possible AEs were identified as combined use of these drugs.

### Statistical analysis

First, to detect the medication patterns in the reports of pregnant women using psychiatric medication, we classified the reports based the names of the drugs and their administration numbers in the reports, combining the two databases using LCA. LCA is one of the methods of cluster analysis that is used to identify natural subgroups in data with closer resemblance between items within a subgroup than between items in different subgroups. This approach identifies the unobservable subgroup (i.e. latent class), which is measured by responses to observed categorical variables. The classes and their profiles are derived from data without information of pre-specified groups such as medications classification [24]. It is a basic assumption of LCA that the observed variables are not highly correlated within the identified subgroups, which is known as local independence [25]. In our analysis, the presence or absence of each drug and the number of drugs used (1, 2, 3, and 4 or more), including non-psychiatric medicines, were employed as variables in the classification model. Note that the drugs were identified by the substance name, which is the name of active ingredient of drug, and were included as variables for the model only when the drug was reported in more than 2% of the patients using psychiatric medications during pregnancy in both databases. The adopted classification model, i.e., the appropriate number of classes, was selected with the lowest Bayesian information criteria [26]. Here, each individual report was assigned to a specific latent class by the highest posterior class-membership probability [27]. Second, after the classification of the medications, the proportions of each class were calculated by country of database (i.e. JADER and FAERS-US separately).

Third, to assess the relationship between reported AEs and medication pattern in pregnancy, we estimated the odds ratio (OR) of AE reports within each class of medication, compared with the reports of other than psychiatric medications used during pregnancy. For this analysis, AEs were categorized based on system organ class (SOC) in MedDRA. The OR of the AE reports for each class relative to reports without psychiatric medication was calculated as OR = *ad*/*bc*, where *a* and *b* were the number of reported cases containing at least one AE included in the SOC and the number of cases reporting only AEs not belonging to the SOC in the class, respectively; *c* and *d* were the number of reported cases containing at least one AE included in the SOC and not belonging to the SOC in the patients without psychiatric medication during pregnancy, respectively. We also provided the 95% confidence interval (CI) of OR and the *p-*values based on a chi-square test without continuity correction. The significance level was set at *p* < 0.05 for all statistical tests. All analyses were performed using SAS 9.4 version (SAS Institute Inc., Cary, NC, USA). LCA was performed using PROC LCA provided by Lanza et al. [28].

## Results

### Classified psychiatric medication patterns

Table 1 summarizes the reports of patients receiving psychiatric medication during pregnancy in the two datasets. Among all reported cases, those from pregnant women were 1.4% and 1.3% for JADER and FAERS-US, respectively. Among pregnant women, patients receiving psychiatric medication totaled 22.0% (1,654/7,530) for JADER and 16.6% (8,735/52,554) for FARES-US. The total number of reported patients using psychiatric medication during pregnancy across both datasets was 10,389 and was further categorized into 11 classes based on medication patterns. Table 2 shows the number and proportion of cases belonging to the classes (class size) in total reports that pooled JADER and FAERS-US in header row, as well as the percentages of each medication and number of exposed drugs within each class; for example, the percentage of lamotrigine in class 1 was 10.5%, which was 2,417 records of class 1 included 254 reports of lamotrigine (254/2,417 = 10.5%).

**Table 1:** Number of cases of adverse events from spontaneous reports

	JADER	FAERS-US
All reported cases	555,301	3,908,398
Reports with any medication in pregnancies	7,530 (1.4%)	52,544 (1.3%)
Reports with psychiatric medications in pregnancies	1,654 (0.3%)	8,735 (0.2%)
Reports with psychiatric medications	108,375 (19.5%)	842,384 (21.6%)

**Table 2:** Class size and profile of classified medication pattern in reports with psychiatric medications during pregnancy

Class	1	2	3	4	5	6	7	8	9	10	11
Class size^a^	23.3%	17.2%	15.2%	11.1%	11.0%	5.7%	4.5%	3.9%	3.6%	2.6%	2.0%
Number of reports	*n*=2417	*n*=1789	*n*=1582	*n*=1153	*n*=1143	*n*=590	*n*=463	*n*=404	*n*=373	*n*=272	*n*=203
**Hypnotics and sedatives, antianxietics**											
Alprazolam	0.8%	13.6%	9.9%	11.4%	0.0%	0.0%	0.0%	0.0%	5.1%	0.0%	2.5%
Lorazepam	0.2%	9.0%	3.2%	11.0%	0.0%	0.0%	4.8%	0.0%	0.0%	5.9%	27.1%
Zolpidem tartrate	0.7%	9.8%	1.6%	14.2%	0.0%	0.0%	0.0%	0.0%	0.5%	0.0%	15.8%
Diazepam	0.5%	4.4%	1.4%	11.7%	0.0%	0.5%	1.3%	0.0%	0.0%	0.0%	2.5%
**Antiepileptics**											
Lamotrigine	10.5%	7.2%	5.1%	0.1%	0.0%	45.1%	11.0%	0.0%	0.0%	0.7%	46.8%
Levetiracetam	7.3%	1.7%	0.0%	0.0%	0.0%	30.3%	61.1%	0.0%	0.3%	2.2%	2.5%
Clonazepam	1.6%	11.0%	6.1%	11.8%	0.0%	8.3%	11.4%	0.0%	1.1%	0.0%	48.8%
Gabapentin	0.0%	18.4%	0.0%	0.0%	0.0%	1.4%	0.0%	0.0%	0.0%	100.0%	1.0%
Topiramate	4.3%	9.8%	1.7%	0.0%	0.0%	0.0%	58.3%	0.0%	6.7%	0.0%	1.0%
Divalproex sodium	11.1%	2.4%	1.3%	1.6%	0.0%	0.5%	3.9%	0.0%	0.3%	0.0%	2.0%
Carbamazepine	2.5%	1.2%	0.0%	5.5%	0.0%	30.5%	7.1%	0.0%	0.0%	0.4%	1.5%
Valproate sodium	3.8%	0.0%	0.0%	6.7%	0.0%	23.2%	2.8%	0.0%	0.0%	0.4%	0.0%
Vigabatrin	4.9%	0.1%	0.0%	0.0%	0.0%	0.0%	19.4%	0.0%	0.0%	0.0%	0.0%
**Antidepressant**											
Paroxetine hydrochloride	0.0%	4.9%	8.2%	18.9%	100.0%	0.7%	0.0%	0.0%	4.6%	0.0%	0.0%
Sertraline hydrochloride	9.9%	9.9%	14.6%	4.0%	0.0%	0.2%	1.5%	0.0%	99.7%	0.4%	21.2%
Duloxetine hydrochloride	12.9%	9.1%	11.6%	0.0%	0.0%	0.0%	0.4%	0.0%	0.0%	0.0%	19.2%
Fluoxetine hydrochloride	0.0%	14.3%	12.4%	2.6%	0.0%	0.0%	0.2%	0.0%	1.3%	0.0%	1.0%
Escitalopram oxalate	3.8%	9.4%	8.5%	1.3%	0.0%	0.0%	0.4%	0.0%	13.7%	0.0%	6.4%
Venlafaxine hydrochloride	1.3%	11.9%	4.0%	0.0%	0.0%	0.0%	1.3%	0.0%	1.6%	0.0%	8.4%
Bupropion hydrochloride	0.0%	9.2%	4.2%	0.0%	0.0%	0.0%	0.0%	0.0%	2.4%	0.4%	13.3%
Trazodone hydrochloride	0.0%	9.7%	2.7%	1.6%	0.0%	0.0%	0.0%	0.0%	0.0%	3.7%	9.4%
**Antipsychotic agent**											
Aripiprazole	0.0%	3.6%	23.6%	1.1%	0.0%	2.2%	0.0%	100.0%	0.0%	0.0%	96.1%
Risperidone	2.9%	1.0%	5.1%	10.7%	0.0%	0.0%	1.1%	0.0%	0.0%	0.7%	24.6%
Olanzapine	5.1%	1.5%	5.2%	4.0%	0.0%	0.0%	0.0%	0.0%	0.3%	0.0%	4.4%
Quetiapine fumarate	0.9%	3.8%	4.0%	7.4%	0.0%	0.0%	0.0%	0.0%	0.5%	0.0%	22.7%
**Other drugs**											
Vitamins	0.0%	25.3%	5.8%	0.0%	0.0%	1.7%	15.8%	0.0%	45.6%	0.0%	7.4%
Folic acid	0.0%	9.7%	0.0%	1.0%	0.0%	26.6%	13.2%	0.0%	5.1%	0.4%	3.0%
Paracetamol	0.0%	17.6%	0.3%	3.3%	0.0%	1.9%	0.9%	0.0%	13.4%	0.0%	0.5%
Oxybate sodium	0.0%	19.4%	2.5%	0.1%	0.0%	0.0%	0.0%	0.0%	0.8%	0.0%	1.0%
Ibuprofen	0.0%	14.5%	0.3%	1.0%	0.0%	0.0%	0.2%	0.0%	10.2%	0.4%	0.0%
Colecalciferol	0.0%	14.6%	0.1%	0.0%	0.0%	0.0%	3.2%	0.0%	5.1%	0.0%	1.0%
Ondansetron hydrochloride	0.0%	11.7%	0.0%	0.1%	0.0%	0.0%	1.1%	0.0%	9.9%	0.4%	13.3%
Levothyroxine sodium	0.0%	9.4%	0.6%	1.8%	0.0%	0.0%	2.6%	0.0%	4.0%	0.0%	5.4%
Prednisone	0.0%	10.6%	0.0%	1.4%	0.0%	0.0%	2.8%	0.0%	3.2%	0.4%	0.0%
Acetaminophen/hydrocodone bitartrate	0.0%	11.7%	0.0%	1.1%	0.0%	0.0%	0.2%	0.0%	1.1%	0.4%	0.0%
**Number of exposed drugs**											
1	98.8%	0.0%	0.2%	0.0%	88.3%	0.0%	0.0%	100.0%	0.0%	12.5%	0.0%
2	1.2%	0.0%	51.8%	0.2%	7.4%	60.2%	14.0%	0.0%	5.1%	27.2%	0.0%
3	0.0%	0.0%	34.8%	8.7%	4.3%	28.3%	27.4%	0.0%	8.3%	27.2%	0.0%
4≤	0.0%	100.0%	13.2%	91.2%	0.0%	11.5%	58.5%	0.0%	86.6%	33.1%	100.0%

^a^The percentage of class size was calculated using total number of reports including JADER and FAERS-US

The percentage of each medication was calculated using number of reports (small n) for each class as denominator

Class 1 was the largest of the 11 classes. Lamotrigine, divalproex sodium or duloxetine hydrochloride were used in over 10% of the patients within class 1, while almost all patients were under monotherapy, using only a single medication. Conversely, all patients in class 2 were receiving four or more drugs as polypharmacy. Class 3 also mainly included patients with two or more drugs including anti-depressant medicines and/or antipsychotic agents. Almost all patients in class 4 were receiving four or more drugs but received fewer non-psychiatric drugs than patients in class 2, such as vitamins and oxybate sodium. In class 5, all patients were receiving paroxetine hydrochloride. Class 6 mainly included patients receiving two or more drugs, including antiepileptic drugs. Although class 7 mainly included patients receiving multiple antiepileptic medications, approximately 60% of them received levetiracetam and topiramate, and the percentage of patients receiving four or more drugs was larger than that in class 6. Almost all patients in classes 8–11 received aripiprazole as a single medication, sertraline hydrochloride in multiple medications, gabapentin and aripiprazole in multiple medications with four or more drugs, respectively.

**Fig. 1:**
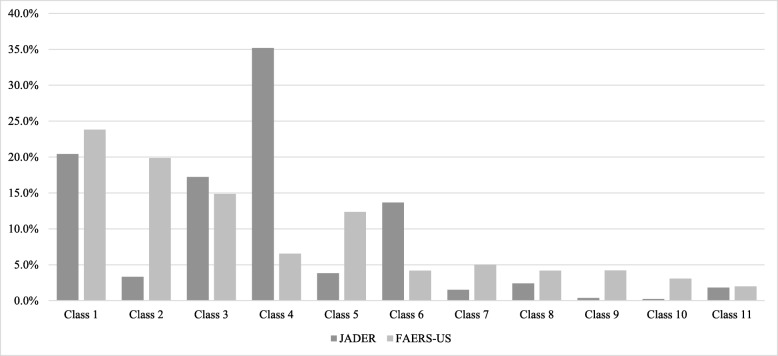
Proportion of class for each medication pattern in JADER and FAERS-US

To compare the composition of patients between Japan and the US, we showed the proportions of classes in each database (Fig. 1). Class 1 comprised 20.4% and 23.8% of JADER and FAERS-US reports, respectively, and was the largest class in FAERS-US and the second largest in JADER. Oppositely, class 4 accounted for the largest percentage of reports in JADER, and class 4 and class 6 accounted for a larger percentage of reports in JADER compared with FAERS-US one. Classes 2, 5, 7, 8, 9, and 10 had a larger percentage in FAERS-US than in JADER.

### OR of AE reports classified by SOC for each medication class

ORs of the AE reports among patients within the class relative to the reports among patients without psychiatric medication during pregnancy are shown in Table 3 (JADER) and Table 4 (FAERS-US). For example, the number and OR of pregnancy reports and perinatal conditions (Preg) were 93 and 0.7 (95% CI = 0.5–0.8) in class 1, respectively. OR in each class was compared with 2,156 reports of Preg within a total of 5,876 reports from pregnancy without the use of psychiatric medications. Note that ORs for AE reports with five cases or fewer are not shown.

**Table 3:** OR of reporting AEs by medication class of pregnancy in JADER, categorized based on system organ class (SOC)

	Class 1 *n*=338	Class 2 *n*=55	Class 3 *n*=285	Class 4 *n*=582	Class 5 *n*=63	Class 6 *n*=226	Class 7 *n*=25	Class 8 *n*=40	Class 9 *n*=6	Class 10 *n*=4	Class 11 *n*=30	Reports in pregnancy without psychiatric medications, *n*=5,876
SOC	# reports OR (95%CI)	# reports OR (95%CI)	# reports OR (95%CI)	# reports OR (95%CI)	# reports OR (95%CI)	# reports OR (95%CI)	# reports OR (95%CI)	# reports OR (95%CI)	# reports OR (95%CI)	# reports OR (95%CI)	# reports OR (95%CI)	n (reference)
Preg	**93**	20	98	**126**	16	73	5	**30**	1	2	**20**	2,156
	**0.7 (0.5,0.8)**	1.0 (0.6,1.7)	0.9 (0.7,1.2)	**0.5 (0.4,0.6)**	0.6 (0.3,1.0)	0.8 (0.6,1.1)	-	**5.2 (2.5,10.6)**	-	-	**3.4 (1.6,7.4)**	1.0
Cong	**135**	3	42	**124**	14	**93**	7	5	0	1	3	1,037
	**3.1 (2.5,3.9)**	-	0.8 (0.6,1.1)	**1.3 (1.0,1.6)**	1.3 (0.7,2.4)	**3.3 (2.5,4.3)**	1.8 (0.8,4.4)	-	-	-	-	1.0
Resp	39	11	**93**	**144**	**19**	**17**	4	4	2	1	6	712
	0.9 (0.7,1.3)	1.8 (0.9,3.5)	**3.5 (2.7,4.6)**	**2.4 (1.9,2.9)**	**3.1 (1.8,5.4)**	**0.6 (0.4,1.0)**	-	-	-	-	1.8 (0.7,4.5)	1.0
Genrl	**37**	**7**	**65**	**169**	**15**	**21**	5	1	2	1	1	252
	**2.7 (1.9,3.9)**	**3.3 (1.5,7.3)**	**6.6 (4.9,8.9)**	**9.1 (7.3,11.4)**	**7.0 (3.9,12.6)**	**2.3 (1.4,3.6)**	-	-	-	-	-	1.0
Nerv	30	4	**37**	**108**	**12**	**23**	3	1	4	0	2	420
	1.3 (0.9,1.9)	-	**1.9 (1.4,2.8)**	**3.0 (2.3,3.7)**	**3.1 (1.6,5.8)**	**1.5 (0.9,2.3)**	-	-	-	-	-	1.0
Psych	**9**	3	**13**	**39**	**7**	**16**	0	3	1	0	2	28
	**5.7 (2.7,12.2)**	-	**10.0 (5.1,19.5)**	**15.0 (9.2,24.6)**	**26.1 (10.9,62.3)**	**15.9 (8.5,29.9)**	-	-	-	-	-	1.0
Inv	**12**	6	23	32	2	4	2	2	0	0	0	389
	**0.5 (0.3,0.9)**	1.7 (0.7,4.1)	1.2 (0.8,1.9)	0.8 (0.6,1.2)	-	-	-	-	-	-	-	1.0
Metab	4	3	10	37	1	8	4	3	1	1	2	316
	-	-	0.6 (0.3,1.2)	1.2 (0.8,1.7)	-	0.6 (0.3,1.3)	-	-	-	-	-	1.0
Inj&P	**8**	4	13	31	0	7	0	0	2	0	0	295
	**0.5 (0.2,0.9)**	-	0.9 (0.5,1.6)	1.1 (0.7,1.6)	-	0.6 (0.3,1.3)	-	-	-	-	-	1.0
Card	**11**	2	2	**29**	2	**6**	2	1	0	0	1	429
	**0.4 (0.2,0.8)**	-	-	**0.7 (0.5,1.0)**	-	**0.3 (0.2,0.8)**	-	-	-	-	-	1.0
Musc	9	2	**18**	17	2	5	2	0	0	0	0	153
	1.0 (0.5,2.0)	-	**2.5 (1.5,4.2)**	1.1 (0.7,1.9)	-	-	-	-	-	-	-	1.0
Gastr	4	2	**6**	22	4	9	1	1	0	0	0	284
	-	-	**0.4 (0.2,1.0)**	0.8 (0.5,1.2)	-	0.8 (0.4,1.6)	-	-	-	-	-	1.0
Surg	**15**	2	6	5	1	**14**	1	1	0	0	3	149
	**1.8 (1.0,3.1)**	-	0.8 (0.4,1.9)	-	-	**2.5 (1.4,4.5)**	-	-	-	-	-	1.0
Blood	0	**7**	3	26	0	5	1	0	0	0	0	339
	-	**2.4 (1.1,5.3)**	-	0.8 (0.5,1.1)	-	-	-	-	-	-	-	1.0
Skin	2	**7**	1	18	1	4	2	0	0	0	0	212
	-	**3.9 (1.7,8.7)**	-	0.9 (0.5,1.4)	-	-	-	-	-	-	-	1.0
Hepat	3	**6**	3	17	0	3	0	1	0	0	0	184
	-	**3.8 (1.6,9.0)**	-	0.9 (0.6,1.5)	-	-	-	-	-	-	-	1.0
Infec	0	2	3	12	1	5	3	0	0	0	1	194
	-	-	-	0.6 (0.3,1.1)	-	-	-	-	-	-	-	1.0
Immun	1	3	0	14	0	0	1	0	0	0	0	172
	-	-	-	0.8 (0.5,1.4)	-	-	-	-	-	-	-	1.0
Eye	2	0	3	6	1	4	0	0	0	0	0	43
	-	-	-	1.4 (0.6,3.3)	-	-	-	-	-	-	-	1.0
Repro	2	0	2	9	1	1	0	1	0	0	0	160
	-	-	-	0.6 (0.3,1.1)	-	-	-	-	-	-	-	1.0
Vasc	2	1	3	11	0	1	0	0	0	0	0	177
	-	-	-	0.6 (0.3,1.1)	-	-	-	-	-	-	-	1.0
Neopl	1	1	0	3	1	4	0	0	0	0	0	46
	-	-	-	-	-	-	-	-	-	-	-	1.0
Endo	1	0	3	3	0	1	0	0	1	0	0	56
	-	-	-	-	-	-	-	-	-	-	-	1.0
Ear	3	0	0	2	1	2	0	0	0	0	0	16
	-	-	-	-	-	-	-	-	-	-	-	1.0
Renal	1	1	1	3	0	1	0	0	0	0	0	167
	-	-	-	-	-	-	-	-	-	-	-	1.0
SocCi	1	0	0	0	0	0	0	0	0	0	0	1
	-	-	-	-	-	-	-	-	-	-	-	1.0
Prod	0	0	0	0	0	0	0	0	0	0	0	5
	-	-	-	-	-	-	-	-	-	-	-	1.0

Significant results with *p*<0.05 in boldface

OR was estimated in cells with > 5 events

*AE* Adverse event, *OR* Odds ratio, *SOC* System organ class, *Blood* Blood and lymphatic system disorders, *Card* Cardiac disorders, *Cong* Congenital, familial and genetic disorders, *Ear* Ear and labyrinth disorders, *Endo* Endocrine disorders, *Eye* Eye disorders, *Gastr* Gastrointestinal disorders, *Genrl* General disorders and administration site conditions, *Hepat* Hepatobiliary disorders, *Immun* Immune system disorders, *Infec* Infections and infestations, *Inj&P* Injury, poisoning and procedural complications, *Inv* Investigations, *Metab* Metabolism and nutrition disorders, *Musc* Musculoskeletal and connective tissue disorders, *Neopl* Neoplasms benign, malignant and unspecified (incl cysts and polyps), *Nerv* Nervous system disorders, *Preg* Pregnancy, puerperium and perinatal conditions, *Prod* Product issues, *Psych* Psychiatric disorders, *Renal* Renal and urinary disorders, *Repro* Reproductive system and breast disorders, *Resp* Respiratory, thoracic and mediastinal disorders, *Skin* Skin and subcutaneous tissue disorders, *SocCi* Social circumstances, *Surg* Surgical and medical procedures, *Vasc* Vascular disorders

**Table 4:** OR of reporting AEs by medication class of pregnancy in FAERS-US, categorized based on system organ class (SOC)

	Class 1 *n*=2,079	Class 2 *n*=1,734	Class 3 *n*=1,297	Class 4 *n*=571	Class 5 *n*=1,080	Class 6 *n*=364	Class 7 *n*=438	Class 8 *n*=364	Class 9 *n*=367	Class 10 *n*=268	Class 11 *n*=173	Reports in pregnancy without psychiatric medications, *n*=43,807
SOC	# reports OR (95%CI)	# reports OR (95%CI)	# reports OR (95%CI)	# reports OR (95%CI)	# reports OR (95%CI)	# reports OR (95%CI)	# reports OR (95%CI)	# reports OR (95%CI)	# reports OR (95%CI)	# reports OR (95%CI)	# reports OR (95%CI)	n (reference)
Preg	**447**	**615**	**549**	**214**	**98**	**189**	**139**	**147**	**145**	**53**	**103**	20,376
	**0.3 (0.3,0.4)**	**0.6 (0.6,0.7)**	**0.8 (0.8,0.9)**	**0.7 (0.6,0.8)**	**0.1 (<0.1,0.1)**	**1.2 (1.0,1.5)**	**0.5 (0.4,0.7)**	**0.8 (0.6,1.0)**	**0.8 (0.6,0.9)**	**0.3 (0.2,0.4)**	**1.7 (1.2,2.3)**	1.0
Cong	**711**	**629**	**319**	**210**	**931**	**88**	65	**33**	**156**	35	29	7,337
	**2.6 (2.4,2.8)**	**2.8 (2.6,3.1)**	**1.6 (1.4,1.8)**	**2.9 (2.4,3.4)**	**31.1 (26.1,37.0)**	**1.6 (1.2,2.0)**	0.9 (0.7,1.1)	**0.5 (0.3,0.7)**	**3.7 (3.0,4.5)**	0.7 (0.5,1.1)	1.0 (0.7,1.5)	1.0
Resp	**61**	**283**	**130**	**117**	**97**	3	**47**	**9**	**43**	23	**23**	3,192
	**0.4 (0.3,0.5)**	**2.5 (2.2,2.8)**	**1.4 (1.2,1.7)**	**3.3 (2.7,4.0)**	**1.3 (1.0,1.6)**	-	**1.5 (1.1,2.1)**	**0.3 (0.2,0.6)**	**1.7 (1.2,2.3)**	1.2 (0.8,1.8)	**2.0 (1.3,3.0)**	1.0
Genrl	**328**	**549**	**304**	165	**103**	**59**	123	**42**	97	**174**	44	12,315
	**0.5 (0.4,0.5)**	**1.2 (1.1,1.3)**	**0.8 (0.7,0.9)**	1.0 (0.9,1.2)	**0.3 (0.2,0.3)**	**0.5 (0.4,0.7)**	1.0 (0.8,1.2)	**0.3 (0.2,0.5)**	0.9 (0.7,1.2)	**4.7 (3.7,6.1)**	0.9 (0.6,1.2)	1.0
Nerv	**459**	**379**	**219**	**129**	**110**	**89**	**215**	24	**44**	**44**	**33**	3,394
	**3.4 (3.0,3.8)**	**3.3 (3.0,3.8)**	**2.4 (2.1,2.8)**	**3.5 (2.8,4.2)**	**1.4 (1.1,1.7)**	**3.9 (3.0,4.9)**	**11.5 (9.5,13.9)**	0.8 (0.6,1.3)	**1.6 (1.2,2.2)**	**2.3 (1.7,3.2)**	**2.8 (1.9,4.1)**	1.0
Psych	**253**	**344**	**250**	**122**	**108**	31	**81**	**40**	**46**	**36**	**46**	3,251
	**1.7 (1.5,2.0)**	**3.1 (2.7,3.5)**	**3.0 (2.6,3.4)**	**3.4 (2.8,4.2)**	**1.4 (1.1,1.7)**	1.2 (0.8,1.7)	**2.8 (2.2,3.6)**	**1.5 (1.1,2.1)**	**1.8 (1.3,2.4)**	**1.9 (1.4,2.8)**	**4.5 (3.2,6.3)**	1.0
Inv	**95**	**277**	**143**	**101**	**130**	24	33	**16**	33	17	**32**	3,502
	**0.6 (0.4,0.7)**	**2.2 (1.9,2.5)**	**1.4 (1.2,1.7)**	**2.5 (2.0,3.1)**	**1.6 (1.3,1.9)**	0.8 (0.5,1.2)	0.9 (0.7,1.3)	**0.5 (0.3,0.9)**	1.1 (0.8,1.6)	0.8 (0.5,1.3)	**2.6 (1.8,3.8)**	1.0
Metab	73	**177**	**76**	**62**	**30**	**6**	**38**	5	21	10	**42**	1,878
	0.8 (0.6,1.0)	**2.5 (2.2,3.0)**	**1.4 (1.1,1.8)**	**2.7 (2.1,3.6)**	**0.6 (0.4,0.9)**	**0.4 (0.2,0.8)**	**2.1 (1.5,3.0)**	-	1.4 (0.9,2.1)	0.9 (0.5,1.6)	**7.2 (5.0,10.2)**	1.0
Inj&P	**1654**	**1170**	**1034**	**423**	**1064**	**269**	251	**324**	**296**	**196**	**136**	25,556
	**2.8 (2.5,3.1)**	**1.5 (1.3,1.6)**	**2.8 (2.4,3.2)**	**2.0 (1.7,2.5)**	**47.3 (28.9,77.4)**	**2.0 (1.6,2.6)**	1.0 (0.8,1.2)	**5.8 (4.2,8.0)**	**3.0 (2.3,3.9)**	**1.9 (1.5,2.5)**	**2.6 (1.8,3.8)**	1.0
Card	**66**	**179**	**86**	**89**	**153**	8	21	2	**55**	**30**	**13**	1,770
	**0.8 (0.6,1.0)**	**2.7 (2.3,3.2)**	**1.7 (1.3,2.1)**	**4.4 (3.5,5.5)**	**3.9 (3.3,4.7)**	0.5 (0.3,1.1)	1.2 (0.8,1.9)	-	**4.2 (3.1,5.6)**	**3.0 (2.0,4.4)**	**1.9 (1.1,3.4)**	1.0
Musc	**43**	**226**	79	**73**	52	12	19	2	**34**	**25**	8	2,430
	**0.4 (0.3,0.5)**	**2.6 (2.2,3.0)**	1.1 (0.9,1.4)	**2.5 (1.9,3.2)**	0.9 (0.6,1.1)	0.6 (0.3,1.0)	0.8 (0.5,1.2)	-	**1.7 (1.2,2.5)**	**1.8 (1.2,2.6)**	0.8 (0.4,1.7)	1.0
Gastr	**145**	**350**	**153**	**91**	**56**	**19**	**73**	3	33	**39**	**33**	4,137
	**0.7 (0.6,0.9)**	**2.4 (2.1,2.7)**	**1.3 (1.1,1.5)**	**1.8 (1.4,2.3)**	**0.5 (0.4,0.7)**	**0.5 (0.3,0.8)**	**1.9 (1.5,2.5)**	-	0.9 (0.7,1.4)	**1.6 (1.2,2.3)**	**2.3 (1.5,3.3)**	1.0
Surg	**36**	**112**	83	39	**10**	**10**	15	27	14	13	**37**	2,348
	**0.3 (0.2,0.4)**	**1.2 (1.0,1.5)**	1.2 (1.0,1.5)	1.3 (0.9,1.8)	**0.2 (<0.1,0.3)**	**0.5 (0.3,0.9)**	0.6 (0.4,1.0)	1.4 (1.0,2.1)	0.7 (0.4,1.2)	0.9 (0.5,1.6)	**4.8 (3.3,6.9)**	1.0
Blood	4	**144**	31	**34**	5	2	10	0	11	9	2	1,458
	-	**2.6 (2.2,3.1)**	0.7 (0.5,1.0)	**1.8 (1.3,2.6)**	-	-	0.7 (0.4,1.3)	-	0.9 (0.5,1.6)	1.0 (0.5,2.0)	-	1.0
Skin	**23**	**126**	46	29	**9**	2	15	1	16	6	0	2,015
	**0.2 (0.2,0.4)**	**1.6 (1.3,2.0)**	0.8 (0.6,1.0)	1.1 (0.8,1.6)	**0.2 (<0.1,0.3)**	-	0.7 (0.4,1.2)	-	0.9 (0.6,1.6)	0.5 (0.2,1.1)	-	1.0
Hepat	**11**	**49**	26	**22**	21	2	3	1	7	2	**15**	657
	**0.3 (0.2,0.6)**	**1.9 (1.4,2.6)**	1.3 (0.9,2.0)	**2.6 (1.7,4.1)**	1.3 (0.8,2.0)	-	-	-	1.3 (0.6,2.7)	-	**6.2 (3.7,10.6)**	1.0
Infec	**38**	**264**	79	**68**	**60**	**8**	33	**8**	**79**	5	15	3,181
	**0.2 (0.2,0.3)**	**2.3 (2.0,2.6)**	0.8 (0.7,1.0)	**1.7 (1.3,2.2)**	**0.8 (0.6,1.0)**	**0.3 (0.1,0.6)**	1.0 (0.7,1.5)	**0.3 (0.1,0.6)**	**3.5 (2.7,4.5)**	-	1.2 (0.7,2.1)	1.0
Immun	3	**48**	11	**13**	3	2	2	1	**23**	0	0	550
	-	**2.2 (1.7,3.0)**	0.7 (0.4,1.2)	**1.8 (1.1,3.2)**	-	-	-	-	**5.3 (3.4,8.1)**	-	-	1.0
Eye	31	**60**	**32**	**24**	24	4	11	2	8	**18**	4	725
	0.9 (0.6,1.3)	**2.1 (1.6,2.8)**	**1.5 (1.1,2.2)**	**2.6 (1.7,3.9)**	1.4 (0.9,2.0)	-	1.5 (0.8,2.8)	-	1.3 (0.7,2.7)	**4.3 (2.6,6.9)**	-	1.0
Repro	**46**	131	88	36	**13**	**7**	**10**	**10**	24	5	6	2,944
	**0.3 (0.2,0.4)**	1.1 (0.9,1.4)	1.0 (0.8,1.3)	0.9 (0.7,1.3)	**0.2 (<0.1,0.3)**	**0.3 (0.1,0.6)**	**0.3 (0.2,0.6)**	**0.4 (0.2,0.7)**	1.0 (0.6,1.5)	-	0.5 (0.2,1.1)	1.0
Vasc	**24**	**144**	61	**55**	30	5	14	1	**22**	8	4	1,675
	**0.3 (0.2,0.4)**	**2.3 (1.9,2.7)**	1.2 (1.0,1.6)	**2.7 (2.0,3.6)**	0.7 (0.5,1.0)	-	0.8 (0.5,1.4)	-	**1.6 (1.0,2.5)**	0.8 (0.4,1.6)	-	1.0
Neopl	**8**	**60**	20	12	10	2	7	0	4	3	2	668
	**0.2 (0.1,0.5)**	**2.3 (1.8,3.0)**	1.0 (0.6,1.6)	1.4 (0.8,2.5)	0.6 (0.3,1.1)	-	1.1 (0.5,2.2)	-	-	-	-	1.0
Endo	18	**40**	9	**9**	3	0	1	0	3	0	3	339
	1.1 (0.7,1.8)	**3.0 (2.2,4.2)**	0.9 (0.5,1.7)	**2.1 (1.1,4.0)**	-	-	-	-	-	-	-	1.0
Ear	17	**44**	14	**11**	**18**	2	4	1	**10**	1	1	422
	0.8 (0.5,1.4)	**2.7 (2.0,3.7)**	1.1 (0.7,1.9)	**2.0 (1.1,3.7)**	**1.7 (1.1,2.8)**	-	-	-	**2.9 (1.5,5.4)**	-	-	1.0
Renal	**10**	**99**	33	**28**	32	4	6	3	11	4	5	1,037
	**0.2 (0.1,0.4)**	**2.5 (2.0,3.1)**	1.1 (0.8,1.5)	**2.1 (1.4,3.1)**	1.3 (0.9,1.8)	-	0.6 (0.3,1.3)	-	1.3 (0.7,2.3)	-	-	1.0
SocCi	**124**	**50**	**38**	8	4	**13**	**10**	**37**	6	4	**8**	517
	**5.3 (4.3,6.5)**	**2.5 (1.9,3.3)**	**2.5 (1.8,3.5)**	1.2 (0.6,2.4)	-	**3.1 (1.8,5.4)**	**2.0 (1.0,3.7)**	**9.5 (6.7,13.5)**	1.4 (0.6,3.1)	-	**4.1 (2.0,8.3)**	1.0
Prod	**11**	59	38	15	2	2	**7**	0	12	1	1	1,627
	**0.1 (<0.1,0.3)**	0.9 (0.7,1.2)	0.8 (0.6,1.1)	0.7 (0.4,1.2)	-	-	**0.4 (0.2,0.9)**	-	0.9 (0.5,1.6)	-	-	1.0

Significant results with *p*<0.05 in boldface

OR was estimated in cells with > 5 events

*AE* Adverse event, *OR* Odds ratio, *SOC* System organ class, *Blood* Blood and lymphatic system disorders, *Card* Cardiac disorders, *Cong* Congenital, familial and genetic disorders, *Ear* Ear and labyrinth disorders, *Endo* Endocrine disorders, *Eye* Eye disorders, *Gastr* Gastrointestinal disorders, *Genrl* General disorders and administration site conditions, *Hepat* Hepatobiliary disorders, *Immun* Immune system disorders, *Infec* Infections and infestations, *Inj&P* Injury, poisoning and procedural complications, *Inv* Investigations, *Metab* Metabolism and nutrition disorders, *Musc* Musculoskeletal and connective tissue disorders, *Neopl* Neoplasms benign, malignant and unspecified (incl cysts and polyps), *Nerv* Nervous system disorders, *Preg* Pregnancy, puerperium and perinatal conditions, *Prod* Product issues, *Psych* Psychiatric disorders, *Renal* Renal and urinary disorders, *Repro* Reproductive system and breast disorders, *Resp* Respiratory, thoracic and mediastinal disorders, *Skin* Skin and subcutaneous tissue disorders, *SocCi* Social circumstances, *Surg* Surgical and medical procedures, *Vasc* Vascular disorders

Regarding the SOC of congenital, familial, and genetic disorders (Cong), the largest number of reports was found in class 1 of JADER. Moreover, the OR of Cong, general disorders and administration site conditions (Genrl), and psychiatric disorders (Psych) were larger than 1.0 relative to the reports in cases without psychiatric medications during pregnancy. For SOCs of Genrl and Psych ORs, similar results were observed in classes 3, 4, 5, and 6. Furthermore, class 1 also had significantly lower ORs for Preg, investigations (Inv), injury, poisoning, procedural complications (Inj&P), and cardiac disorders (Card), whereas significantly higher ORs were found for surgical and medical procedures (Surg).

Class 2 had significantly higher ORs for some SOCs, but the number of reported AE cases was less than 10. For class 3, significantly higher ORs were observed for respiratory, thoracic, and mediastinal disorders (Resp), Genrl, nervous system disorders (Nerv), Psych, and musculoskeletal and connective tissue disorders (Musc). The maximum number of cases was observed for Preg, but its OR was not significant.

Class 4 had ORs significantly higher than 1 for Cong, Resp, Genrl, Nerv, and Psych. Particularly, the largest SOC was Genrl, which was higher than those of the other classes. Oppositely, class 4 had significantly lower OR for Preg. The ORs of class 5 were significantly higher than 1 for Resp, Genrl, Nerv, and Psych. Class 6 also had significantly higher ORs for Cong and had higher ORs for Genrl and Psych, as well as for Surg. However, for Resp, a significantly lower OR was observed in class 6, in contrast to classes 3, 4, and 5. For classes 7, 9, and 10, the number of cases for all SOCs was lower than 10 without significant ORs. Moreover, for classes 8 and 11, ORs only for Preg were all statistically higher than 1.

In the results from the FAERS-US dataset, class 1 had significantly higher ORs for Cong, Nerv, Psych, Inj&P, and social circumstances. Significantly lower ORs were observed for the other 15 SOCs. For Genrl, OR was 0.5 (95% CI = 0.4–0.5), though it was 2.7 (95% CI = 1.9–3.9) in JADER. The maximum number of reports for Inj&P were observed for all classes. In classes 2 and 4, almost all SOCs had significantly higher ORs, but only Preg had a significantly lower OR. In class 3, in addition to that, Genrl also showed a significantly lower OR. In class 5, the maximum OR was indicated for Inj&P and the second highest for Cong. In class 6, only three cases were reported for Resp and did not indicate statistically significant ORs for Preg and Psych among the 11 classes.

Classes 7, 9, and 10 had some SOCs with significantly higher or lower ORs, unlike the result seen in JADER. In class 8, the OR was significantly lower than 1 for Preg and higher than 1 (OR = 5.2; 95% CI = 2.5–10.6) for JADER. Class 11 showed significantly higher OR for Preg in FAERS-US.

## Discussion

According to data collected from 2011 to 2014 in the Japan Environment and Children’s Study [29], the prevalence of pregnant women who had used one or more drug(s) from the time of pregnancy confirmation until week 12 of pregnancy was over 57% in Japan, whereas the percentage of drug usage excluding iron, folic acid, and other vitamin and mineral supplements was lower at 36%. The prevalence of pregnant women who had used drugs during the first trimester was similar to that reported in studies conducted in the US (39%) [30]. Furthermore, according to a database of consultations during the period from 2005 to 2013 designed by the Japan Drug Information Institute in Pregnancy, almost 50% of women who sought counseling on some kind of medication during pregnancy was doing so regarding psychiatric medication [23]. In this study, we summarized AE cases from databases of spontaneous reports of use of psychiatric medications during pregnancy. Although information regarding the number of patients taking medications was not available via the spontaneous reports, we found that the proportions of AE reports of psychiatric medication during pregnancy among AE reports of all medication during pregnancy were 22.0% and 16.6% in JADER and FAERS-US, respectively. These values were close to or less than the proportions of cases receiving psychiatric medication among all cases regardless of pregnancy (19.5% for JADER and 21.6% for FARES-US). Thus, the proportion of AE reports of psychiatric medication in pregnancy remain the same, and are similar in both countries.

We analyzed spontaneous AE reports in JADER and FAERS-US to identify the medication patterns in pregnancy with psychiatric drugs. Apart from the subgroups detected as latent or unobserved subgroup, we were able to interpret the population of identified medication patterns based on the proportions of each item as shown in Table 2. We were able to identify subgroups including polypharmacy of several psychiatric medications. The medication patterns might also reflect not only the baseline disease, but also type of symptoms and severity of disease.

Moreover, we analyzed the association between AEs and medication patterns in pregnancy with psychiatric drugs. The percentage of specific AE was defined as the impact of this AE relative to other AEs of the drugs. Note that we were unable to estimate the risk of AE since the information on the number of patients receiving each medication was not available in the spontaneous data used. Therefore, signal detection methods [12] were applied to spontaneous reporting data and a drug and event combination table was created. However, in the case of AEs during pregnancy, the number of reported events and medications were often small. We analyzed over 45,000 reports of all medications used during pregnancy and could evaluate reports comparing AEs and medication patterns by classifying the reports using LCA. LCA allowed us to assess relationships between AEs and drug use as well as consider rare events such as congenital anomalies and concomitant medications. Our analysis showed that several patterns of psychiatric medications in pregnancy relate closely to AE reports. This study may provide a possible forecast of the likely AEs of each medication pattern.

The largest class, class 1, mostly consisted of patients under monotherapy, half of patients received antiepileptic drugs, and many reports of Cong were observed. Although the risk of congenital anomalies of the patients depending on the presence or absence of antiepileptic drug use could not be evaluate in this analysis, proportion of the reports of Cong in pregnancy receiving any antiepileptic drugs within class 1 (83/135 = 61.5%) was higher than the reports of Cong in pregnancy without antiepileptic drugs within class 1 (52/203 = 25.6%). OR of Cong in pregnancy receiving any antiepileptic drugs was higher than 1.0 (OR = [83 ×151]/[52 ×52] = 4.6; 95% CI = 2.9–7.4) considering the patients not receiving antiepileptic drugs within class 1. Main reports of antiepileptic drugs and the associated reports were Cong in this class. Although the risk of AE was not assessed in our analysis, the risk of congenital anomalies should be minimized based on several previous studies including risk evaluation of congenital anomalies for several antiepileptic drugs [23, 31]. Conversely, for class 2, all reports included four or more medications as polypharmacy including many non-psychiatric medications, and this class included the largest number of AEs across several SOCs. Patients in class 2 might have several complications and/or severe disease and were mostly contained in the FAERS-US dataset. Reports of class 3 and 4 included multiple psychiatric medications. Although the proportion of class 3 patient reports in each database were similar, the proportion of class 4 patient reports in JADER was higher than in FAERS-US. The proportion of concomitant medication with antidepressant and antianxiety in Japan was higher than that in the US [9]. In fact, the Ministry of Health, Labor, and Welfare of Japan is revising the clinical fee remuneration in order to reduce health insurance reimbursement of medical facilities when more than three psychiatric drugs are prescribed simultaneously, to enhance polypharmacy control measures [32, 33]. In particular, for pregnant women, this is important not only for the evaluation of the patterns of exposure to medication but also to assess the association between AEs and medication patterns in polypharmacy since placental-mediated interactions are possible [34].

ORs for Genrl in almost all classes were higher than 1 in JADER. The largest reports in Genrl were drug withdrawal syndromes in neonates. Neonatal drug withdrawal syndrome has previously been associated with psychiatric medications including anti-epileptics, anti-depressants, anti-anxiety drugs, and anti-psychotic agents [35]. In particular, class 4 in JADER, the OR of Genrl was high within the class and compared with other classes. Class 4 patients received multiple medications (mean number of drugs = 7.6, in contrast with class 3 = 2.8), and especially this class had many patients receiving hypnotics, sedatives, and antianxietics, (mean of number of drugs per patients for class 4 = 1.6, in contrast with class 3 = 0.8). Medication dose is correlated with risk factors in neonatal withdrawal syndrome [36], and the number of different medications used might correlate with disease severity and the total dose of medication in the imipramine-equivalent dose [37]. On the other hand, ORs for Genrl in FAERS-US were lower than those in JADER. This may have occurred because almost all reports of Genrl were from pregnancies without psychiatric medications in FAERS-US, in contrast with the 56% of reports of Genrl that were from pregnancies with psychiatric medication in JADER.

Class 5 had a higher OR of Cong than 1 in FAERS-US, which in turn was higher than that in JADER. This may have occurred because in 2005, the FDA cautioned that exposure to paroxetine during the first trimester of pregnancy may increase the risk of cardiac malformations [38]; this information was not divulged in Japan. Recently, a meta-analysis study reported a generally small risk of congenital malformations of selective serotonin reuptake inhibitors in pregnancy [39]. Although the OR of Cong in class 6, which included antiepileptic drugs with multiple medications, was higher than 1 in both datasets, it was not higher than that of class 1. Female patients with epilepsy are recommended to take folic acid [40]. Furthermore it has been shown that, as a part of antiepileptic combination therapy, co-administration might not further increase the risk of congenital anomalies [41].

In class 7 to class 11, a small number of reports especially of sertraline hydrochloride (class 9) and gabapentin (class 10) were included in JADER, and these were rarer than FAERS-US reports. This indicated that the number of medication patterns might be smaller in JADER than in FAERS-US. This finding might be related to the approval period during which sertraline hydrochloride and gabapentin were introduced in the market, as these drugs were approved later in Japan than in the US. Additionally, the different medication patterns between countries might be influenced by the FDA medication classification of these drugs prior to 2015, which implies a lower risk than other drugs (e.g. paroxetine hydrochloride and valproate sodium) [42]. These differences may aid in evaluating AEs during pregnancy due to psychiatric treatment.

Some AE reports are influenced by the regulations in Japan versus those of the US regardless of the relationship between patterns of psychiatric medications used during pregnancy. For example, the FAERS-US includes ‘drug exposure during pregnancy’ and ‘no adverse events’ based on regulations; such regulations are not mandatory in Japan [19], but a high number of Inj&P and Genrl were reported in FAERS-US.

Our study had several limitations. We could not account for detailed characteristics of patients such as weight, height, primary disease, and information on treatment exposure (exposure period of pregnancy, daily total dose or cumulative dose) of pregnant women, nor on the sex, weight, and height of neonates since this information was not available in the databases. The multiple regression for the adjustment of confounders between each medication pattern and reference group were not performed in the presented analysis. In spontaneous reports, it is important to collect information regarding detecting pregnancy and who is experiencing the AE (i.e. pregnant women or neonates).

## Conclusions

Our analysis showed several patterns of psychiatric medications in pregnancy closely associated with AE. This study may provide a prediction of likely AEs of each medication pattern. Although further research on additional datasets is required to clarify these results, our findings on the association between AE reports and medication patterns could help improve the administration of psychiatric medications during pregnancy. Simultaneously, the proportion of patients receiving four or more psychiatric drugs in JADER was greater than that in FAERS-US, and the classes of medication patterns were fewer in JADER than in FAERS-US, which may affect AE reports in each country. These differences may be useful information in evaluating AEs during pregnancy due to psychiatric medications.

## Supplementary information

**Additional file 1: Table S1.** List of SMQs to select the candidate reports.

## Data Availability

The datasets generated during and/or analyzed during the current study are available in the web site of the Pharmaceuticals and Medical Devices Agency (PMDA), www.pmda.go.jp, and the FDA website, www.fda.gov.

## Abbreviations

AE: Adverse events
Card: Cardiac disorders
Cong: Congenital, familial and genetic disorders
FAERS-US: Food and Drug Administration Adverse Event Reporting System in the US
Genrl: General disorders and administration site conditions
Inj&P: Injury, poisoning and procedural complications
Inv: Investigations
JADER: Japanese Adverse Drug Event Report
LCA: Latent class analysis
MedDRA: Medical dictionary for regulatory activities
Musc: Musculoskeletal and connective tissue disorders
Nerv: Nervous system disorders
OR: Odds ratio
Preg: Pregnancy, puerperium and perinatal conditions
Psych: Psychiatric disorders
Resp: Respiratory, thoracic and mediastinal disorders
SMQs: Standardized MedDRA queries
SOC: System organ class
Surg: Surgical and medical procedures
