# Supplementary material for: Adverse event reports in patients taking psychiatric medication during pregnancy from spontaneous reports in Japan and the United States: an approach using latent class analysis
Source: BMC Psychiatry. 2020 Mar 12;20:118. doi: 10.1186/s12888-020-02525-z (PMC7068895; doi:10.1186/s12888-020-02525-z)
Supplement: Supplementary file 1 — Additional file 1: Table S1. List of SMQs to select the candidate reports. [file 12888_2020_2525_MOESM1_ESM.docx]

**Table S1: List of SMQs to select the candidate reports**

| SMQ code | SMQ names |
| --- | --- |
| 20000186 | Pregnancy, labour and delivery complications and risk factors (excluding abortions and stillbirth) (SMQ) |
| 20000190 | Foetal disorders (SMQ) |
| 20000193 | Normal pregnancy conditions and outcomes (SMQ) |
| 20000077 | Congenital, familial and genetic disorders (SMQ) |
| 20000191 | Neonatal disorders (SMQ) |
| 20000192 | Termination of pregnancy and risk of abortion (SMQ) |

Candidate reports for pregnant woman were selected the reports which 'reported event' were belonging to any of the listed six groups, or 'disease' or 'indication for use' were belonging to any of the first three SMQs.
